# Early neurodevelopmental outcomes of extreme preterm infants exposed to paracetamol: a retrospective cohort study

**DOI:** 10.1038/s41390-023-02649-4

**Published:** 2023-05-17

**Authors:** Bella Zhong, Kenneth Tan, Abdul Razak, Vathana Sackett, Catherine Machipisa, Lindsay Zhou, Samira Samiee-Zafarghandy, Arvind Sehgal, Rod W. Hunt, Pramod Pharande, Atul Malhotra

**Affiliations:** 1 Department of Paediatrics, Monash University, Melbourne, VIC Australia; 2 Monash Newborn, Monash Children’s Hospital, Melbourne, VIC Australia; 3 The Ritchie Centre, Hudson Institute of Medical Research, Melbourne, VIC Australia; 4 Allied Health Department, Monash Children’s Hospital, Melbourne, VIC Australia; 5 Neonatal Division, McMaster University, Hamilton, ON Canada

## Abstract

**Background:**

Paracetamol is commonly used for analgesia and patent ductus arteriosus (PDA) treatment in preterm infants. We aimed to evaluate early neurodevelopmental outcomes of extreme preterm infants exposed to paracetamol during their neonatal admission.

**Methods:**

This retrospective cohort study included surviving infants born at <29 weeks gestation, or with a birth weight of <1000 grams. Neurodevelopmental outcomes studied were early cerebral palsy (CP) or high risk of CP diagnosis, Hammersmith Infant Neurological Examination (HINE) score and Prechtl General Movement Assessment (GMA) at 3–4 months corrected age.

**Results:**

Two hundred and forty-two infants were included, of which 123 were exposed to paracetamol. After adjusting for birth weight, sex and chronic lung disease, there were no significant associations between paracetamol exposure and early CP or high risk of CP diagnosis (aOR 1.46, 95% CI 0.61, 3.5), abnormal or absent GMA (aOR 0.82, 95% CI 0.37, 1.79) or HINE score (adjusted β −0.19, 95% CI −2.39, 2.01). Subgroup analysis stratifying paracetamol exposure into <180 mg/kg or ≥180 mg/kg cumulative dose found that neither had significant effects on outcomes.

**Conclusions:**

In this cohort of extreme preterm infants, no significant association was found between exposure to paracetamol during the neonatal admission and adverse early neurodevelopment.

**Impact:**

- Paracetamol is commonly used in the neonatal period for analgesia and patent ductus arteriosus treatment in preterm infants, although prenatal paracetamol use has been associated with adverse neurodevelopmental outcomes.
- Exposure to paracetamol during the neonatal admission was not associated with adverse early neurodevelopment at 3–4 months corrected age in this cohort of extreme preterm infants.
- The findings from this observational study is consistent with the small body of literature supporting the lack of association between neonatal paracetamol exposure and adverse neurodevelopmental outcomes in preterm infants.

## Introduction

Paracetamol use in the neonatal intensive care unit has increased steadily in the last decade. A French study, which included nearly 30,000 infants over 2017–2018, found that paracetamol was the third most commonly administered medication in neonatal units and was given to more than 65% of all infants born less than 27 weeks gestation.^1^ In addition to its favourable analgesic effects, the increasing use of paracetamol in preterm infants is driven by its use for treating the haemodynamically significant patent ductus arteriosus (PDA).^2,3^ A 2020 Cochrane review concluded that paracetamol has similar efficacy (albeit low- to moderate-quality evidence) and an excellent short-term biochemical and clinical safety profile compared to non-steroidal anti-inflammatory drugs, such as indomethacin and ibuprofen, for PDA treatment.^2^

However, the neurodevelopmental impacts and long-term safety of paracetamol use in preterm infants is still being established. Paracetamol use in pregnancy has been associated with neurocognitive impairments such as attention-deficit hyperactivity disorder symptoms^4,5^ and autism spectrum disorder.^6^ A review of cohort studies of prenatal paracetamol exposure and child neurodevelopment demonstrated that the longer the duration of paracetamol use, the higher the risk of adverse neurodevelopmental outcomes (attention-deficit hyperactivity disorder, autism spectrum disorder, or lower IQ).^7^ Although these studies are observational and retrospective, the use of paracetamol during pregnancy has been cautioned.^8^

This raises the question of whether the concern regarding prenatal exposure can be extrapolated to early neonatal paracetamol use in preterm neonates, especially those born extremely preterm. In the neonatal period, paracetamol exposure during a critical phase of brain development induced long-lasting effects on cognitive function in mice.^9^ An ecological study in the United States found that male circumcision, for which paracetamol is widely used in the neonatal period, strongly correlated with male autism spectrum disorder prevalence between 1995 to 2005.^10^ There is limited data from randomised trials about the neurodevelopmental outcomes of administering paracetamol to preterm infants. Juujärvi et al reported two- and five-year follow up of 44 very preterm infants from their randomised trial of intravenous paracetamol compared to placebo for PDA.^11^ Using the Griffith Mental Development Scales, they found no differences in neurodevelopmental outcomes in their study population.^12,13^ Another small study of 61 children reported no difference in neurodevelopmental outcomes in those exposed to paracetamol compared to ibuprofen for PDA using the Bayley Scales of Infant Development (BSID-II) at two years of age.^14^ A Finnish cohort study of 304 very preterm (<32 weeks) neonates admitted to the neonatal intensive care unit found no significant difference in speech/language developmental disorders, autism spectrum disorder or cerebral palsy (CP) diagnoses in those treated with intravenous paracetamol for any indication compared to those who were not exposed to paracetamol, after five years of follow-up.^15^ However, in view of concerns raised regarding neurodevelopmental outcomes following prenatal and postnatal exposure to paracetamol, there is a pressing need for more neurodevelopmental and long-term follow-up studies of paracetamol use in the newborn population.^2,3,16^

While paracetamol may be used for a variety of indications, its use for PDA treatment is the primary reason in our unit. Open label, first line paracetamol therapy has been the standard of care for medical closure of PDA since 2017. The unit routinely stratifies hemodynamically significant PDA using a functional echocardiographic staging, with an overall treatment rate for PDA in preterm infants less than 32 weeks gestation age around 14%.^17^ Our unit has an Early Neurodevelopment Clinic, which assesses high-risk preterm and term infants for early features of CP and developmental delay at 3–4 months corrected age.^18^ This clinic is based on well-established best practice evidence which shows that standardised clinical assessments done at that age are highly predictive of long-term motor and developmental outcomes, including CP.^19^

Accordingly, we aimed to evaluate the early neurodevelopmental outcomes of extreme preterm infants exposed to paracetamol during their neonatal admission compared to those preterm infants who were not exposed to paracetamol. We hypothesised that preterm infants exposed to paracetamol have similar early neurodevelopmental outcomes to those not treated with paracetamol, given its safety profile.

## Methods

### Setting

A retrospective cohort study was conducted at Monash Children’s Hospital, Melbourne, a perinatal and surgical tertiary neonatal unit providing care to sick preterm and term neonates. All infants born less than 29 weeks gestation or with a birthweight less than 1000 grams are routinely followed up at 3–4 months corrected age (12–14 weeks post-term corrected age) at the Early Neurodevelopment Clinic. A Prechtl General Movement Assessment (GMA) at the fidgety age, and Hammersmith Infant Neurological Examination (HINE) are performed on all infants attending the clinic.^18^ We continued the Early Neurodevelopment Clinic assessments face-to-face during COVID-19 pandemic except during the early lockdown period. Telehealth assessments were done in 10 infants during the study period.

### Eligibility

Infants were eligible to be included in this cohort study if they were born at less than 29 weeks gestation or had a birthweight less than 1000 grams, and survived and attended the 3–4 month follow up Early Neurodevelopmental Clinic. Potential participants were excluded if they did not receive early neonatal care at Monash Children’s Hospital, primarily due to the inability to verify paracetamol exposure in the neonatal admission.

### Outcomes

The primary neurodevelopmental outcome was an early CP diagnosis or high risk of CP diagnosis, at 3–4 months corrected age. The two secondary outcomes were the neurodevelopmental assessments of HINE score and GMA result, also measured at 3–4 months corrected age. Neurodevelopmental assessments were completed by experienced paediatric physiotherapists/occupational therapists and neonatologists. The diagnosis of CP or high risk of CP was based on abnormalities of GMA, HINE and medical assessment. GMA was conducted through observation for fidgety movements, with results classified as either present, abnormal or absent.^20^ The HINE was performed according to the prescribed examination items to generate a global score ranging from 0 to 78, with a score less than 57 considered suboptimal at this age.^21^

Subgroup analysis was conducted with paracetamol exposure stratified into two levels: overall exposure <180 mg/kg and exposure ≥180 mg/kg in the neonatal admission. This was based on a standard dose of paracetamol for PDA for one course (15 mg/kg four times a day for three days).

### Data collection

Data were collected for all eligible infants who attended the Early Neurodevelopmental Clinic between May 2019 and April 2022. This period spanned from the formal establishment of the clinic to the time of data collection and thus included the maximum number of eligible infants. Data for demographic characteristics, paracetamol exposure and potential confounding variables (neonatal morbidities) were retrieved from scanned medical records as well as electronic medical records, for the period of the participant’s initial neonatal admission. Three- to four-month neurodevelopmental outcome data was collected from electronic clinic notes.

### Statistics

Statistical analyses were performed using Stata 16.^22^ Descriptive statistics were used to summarise baseline clinical characteristics. Normality of data was confirmed for data using the Shapiro-Wilk test, and data presented accordingly. The characteristics and outcomes of the infants in each group were compared using the independent *t*-test or the Mann–Whitney *U* test for continuous variables and the chi-square test or Fisher’s exact test for categorical variables, as appropriate. Simple and multivariable linear and logistic regression analyses were used to estimate the association between paracetamol exposure and each of the neurodevelopmental outcomes of early CP/high risk of CP diagnosis, abnormal/absent fidgety GMA, and HINE score, adjusting for confounders such as birth weight, sex, and chronic lung disease. The low frequency of the outcomes limited the number of potential confounders for which we could adjust. Therefore, we prioritised selecting perinatal variables which were significantly different between groups and included chronic lung disease as a neonatal variable that may be present from birth and that can impact neurological development.^23^ The effect of different levels of cumulative paracetamol exposure (<180 mg/kg or ≥180 mg/kg) on early CP/high risk of CP diagnosis, abnormal/absent fidgety GMA, and HINE score were also analysed using simple and multiple linear regression models, adjusting for the same confounders. A *p*-value of <0.05 was considered statistically significant.

## Results

Two hundred and forty-nine infants who were born at <29 weeks or were born weighing <1000 g attended the Early Neurodevelopmental Clinic during the study period. Of these, 242 received neonatal care at Monash Health, and were included in analysis. Table 1 displays the characteristics of included participants. Paracetamol was administered to 123 infants, with cumulative exposure ranging from 7.5 to 957.5 mg/kg. The group exposed to paracetamol was younger (26.2 weeks vs 27.8 weeks), smaller at birth (787.8 g vs 954.8 g), and had more males (52.8% vs 39.5%). Similarly, the infants exposed to paracetamol had more diagnoses of neonatal morbidities (PDA, chronic lung disease, culture-proven sepsis, necrotising enterocolitis, retinopathy of prematurity, severe intraventricular haemorrhage). Seventy-five infants in the paracetamol exposure group (75/123) had a haemodynamically significant PDA. The median postmenstrual age at first paracetamol exposure was 27.6 weeks (IQR 25.4, 33.3).

**Table 1:** Participant demographics.

Patient characteristic	Paracetamol exposure (*N* = 123)	No paracetamol exposure (*N* = 119)	*p*-value
Perinatal variables			
Maternal age (years), median (IQR)	32.0 (30.0, 36.0)	32.0 (29.0, 36.0)	0.94
Gestational age at birth (weeks), mean (SD)	26.2 (1.9)	27.8 (1.7)	<0.001
Male sex	65 (52.8%)	47 (39.5%)	0.03
Birth weight (grams), mean (SD)	787.8 (232.6)	954.8 (253.1)	<0.001
Fetal growth restriction	44 (35.8%)	44 (37.0%)	0.85
Model of delivery			
Vaginal	41 (33.3%)	38 (31.9%)	
Caesarean	82 (66.7%)	81 (68.1%)	0.82
Neonatal variables			
Patent ductus arteriosus	75 (61.0%)	1 (0.8%)	<0.001
Chronic lung disease	101 (82.1%)	59 (49.6%)	<0.001
Postnatal steroid treatment	44 (35.8%)	6 (5.0%)	<0.001
Discharged on home oxygen	31 (25.2%)	8 (6.7%)	<0.001
Sepsis (culture proven)	55 (44.7%)	21 (17.6%)	<0.001
Necrotising enterocolitis ≥ Bell’s Stage II	14 (11.4%)	0 (0.0%)	<0.001
Necrotising enterocolitis requiring surgery	8 (6.5%)	0 (0.0%)	0.005
Any surgery other than for necrotising enterocolitis	31 (25.2%)	4 (3.4%)	<0.001
Hernia repair	23 (18.7%)	3 (2.5%)	<0.001
Laparotomy	11 (8.9%)	1 (0.8%)	0.004
Stoma creation	8 (6.5%)	1 (0.8%)	0.02
Retinopathy of prematurity requiring laser	21 (17.1%)	4 (3.4%)	<0.001
Hearing loss	3 (2.4%)	0 (0.0%)	0.08
Severe intraventricular haemorrhage (Grade III-IV)	13 (10.6%)	2 (1.7%)	0.004
Ventricular dilatation needing shunt/reservoir	3 (2.4%)	0 (0.0%)	0.08
Postmenstrual age at first paracetamol exposure, median (IQR)	27.6 (25.4, 33.3)	-	-

Table 2 presents primary and secondary neurodevelopmental outcomes at clinic attendance at 3–4 months corrected age. Overall, 30 infants had early features of CP or at high risk of CP diagnosis, and 36 infants had an abnormal or absent fidgety GMA outcome. In initial unadjusted analysis as well as after adjusting for birth weight, sex, and chronic lung disease, there were no significant associations found between paracetamol exposure and early CP or high risk of CP diagnosis (OR 2.12, 95% CI 0.95, 4.74; aOR 1.46, 95% CI 0.61, 3.5), and abnormal or absent fidgety GMA (OR 1.46, 95% CI 0.70, 3.04; aOR 0.82, 95% CI 0.37, 1.79). The median HINE score in the exposed group (55.0 [49.8, 60.0]) was lower than that of the unexposed group (58.5 [50.5, 63.0]), and fell in the suboptimal range. Unadjusted analysis showed a significant reduction in HINE score with paracetamol exposure (unadjusted β −4.04, 95% CI −7.06, −1.02). However, the adjusted analysis suggested no association between paracetamol exposure and HINE score (adjusted β −0.19, 95% CI −2.39, 2.01).

**Table 2:** Early neurodevelopmental outcomes in babies exposed to paracetamol versus of those not exposed.

Outcomes	Paracetamol exposure (*N* = 123) *n* (%) or median (IQR)	No paracetamol exposure (*N* = 119) *n* (%) or median (IQR)	Unadjusted risk estimate (95% confidence interval)^a^	Adjusted risk estimate (95% confidence interval)^a,b^
Early features/ high risk of cerebral palsy	20 (16.3%)	10 (8.4%)	2.12 (0.95, 4.74)	1.46 (0.61, 3.50)
Abnormal/absent fidgety GMA	20 (16.3%)	16 (13.4%)	1.46 (0.70, 3.04)	0.82 (0.37, 1.79)
HINE score	55.0 (49.8, 60.0)	58.5 (50.5, 63.0)	-4.04 (-7.06, -1.02)	-0.19 (-2.39, 2.01)

*n* = events, *N* = total patients, ^a^odds ratio for dichotomous outcomes and regression coefficient for continuous outcomes, ^b^birth weight, sex and chronic lung disease were the covariates included in the regression analysis.

**Fig. 1:**
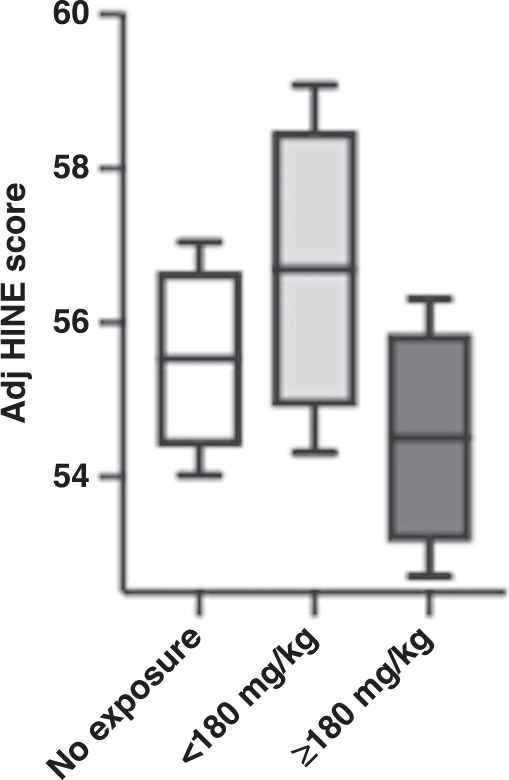
Adjusted mean HINE scores and paracetamol exposure. Estimates of marginal effects by exposure groups (box-whisker plot): adjusted mean HINE score +/− SEM, 95% CI levels. x-axis: paracetamol exposure subgroups; y-axis: mean HINE scores adjusted for sex, birthweight and bronchopulmonary dysplasia.

Subgroup analysis of outcomes in those exposed to <180 mg/kg and ≥180 mg/kg cumulative dose of paracetamol is presented in Table 3. Of those treated with paracetamol, two-thirds had cumulative paracetamol exposure ≥180 mg/kg. In unadjusted analysis, infants with paracetamol exposure ≥180 mg/kg had a significantly higher risk of an early CP or high risk of CP diagnosis (OR 2.64, 95% CI 1.13, 6.17) and lower HINE score (unadjusted β −4.33, 95% CI −7.70, −0.95), while all other associations were insignificant. Results were then adjusted for the same variables of birth weight, sex, and chronic lung disease. This again demonstrated that neither <180 mg/kg nor ≥180 mg/kg cumulative exposure to paracetamol had a significant effect on early CP or high risk of CP diagnosis, abnormal or absent fidgety GMA, nor HINE score. Fig. 1 shows adjusted mean HINE scores according to paracetamol exposure.

**Table 3:** Subgroup analysis of early neurodevelopmental outcomes in babies exposed to high or low cumulative dose of paracetamol.

Outcomes	Paracetamol exposure n/N (%) or median (IQR)	Unadjusted risk estimate (95% confidence interval)^a^	Adjusted risk estimate (95% confidence interval)^a,b^
Early features/high risk of cerebral palsy			
<180 mg/kg	4/41 (9.8%)	1.18 (0.35, 3.98)	0.88 (0.25, 3.12)
≥180 mg/kg	16/82 (19.5%)	2.64 (1.13, 6.17)	1.79 (0.71, 4.52)
Abnormal/absent fidgety GMA			
<180 mg/kg	3/41 (7.3%)	0.81 (0.25, 2.62)	0.37 (0.10–1.42)
≥180 mg/kg	17/82 (20.7%)	1.82 (0.83, 3.97)	1.07 (0.47–2.44)
HINE score			
<180 mg/kg	55.5 (53.0, 61.5) (*N* = 41)	−3.46 (−7.72, 0.80)	0.24 (−2.64, 3.13)
≥180 mg/kg	55.0 (48.5, 60.0) (*N* = 82)	−4.33 (−7.70, −0.95)	−1.41 (−3.94, 1.11)

*n* = events, *N* = total patients, ^a^odds ratio for dichotomous outcomes and regression coefficient for continuous outcomes, ^b^birth weight, sex and chronic lung disease were the covariates included in the regression analysis.

## Discussion

Our study evaluated the early neurodevelopmental outcomes of surviving extremely preterm infants exposed to paracetamol in the neonatal period compared to those who were not exposed to paracetamol. From the spectrum of neurodevelopmental outcomes, the primary outcome examined in this study was early CP or high risk of CP diagnosis, with secondary outcomes being the neurodevelopmental predictors of HINE score, and fidgety GMA result. These were all assessed at 3–4 months corrected age. We found no significant difference between these three early neurodevelopmental outcomes in preterm infants exposed to paracetamol compared to those not treated with paracetamol, after adjusting for birth weight, sex, and chronic lung disease. Additionally, we found no significant associations between paracetamol exposure and early neurodevelopmental outcomes when the exposure was stratified into groups of <180 mg/kg and ≥180 mg/kg cumulative dose.

To our knowledge, this is the first study to examine the association between paracetamol exposure and early neurodevelopmental outcomes in extremely preterm infants at high risk of CP and developmental delay. The early neurodevelopmental assessments performed in this period have been shown to predict long-term neurodevelopmental outcomes such as CP in extremely preterm infants.^19^ This is also one of the largest studies on neurodevelopmental outcomes in extremely preterm infants following paracetamol exposure in the neonatal period.

We found four previous studies which have investigated this subject, three of which were follow-up studies of randomised controlled trials: one by Oncel et al that included 61 preterm infants (≤30 weeks GA) and compared paracetamol to ibuprofen,^14^ and two by Juujärvi et al that included 44 very preterm infants (23–32 weeks GA) and compared paracetamol to placebo.^12,13^ A previous cohort study by Juujärvi et al included 304 very preterm (<32 weeks) neonates and compared paracetamol exposure to no exposure.^15^ These studies also found no differences in neurodevelopmental outcomes between the paracetamol and comparison groups. Our findings are in keeping with these previous studies, although direct comparison is not possible as these previous studies examined outcomes at 18 months to five years. Additionally, these studies used different developmental assessment tools designed for these age groups, such as the Griffith Mental Development Scales and the Bayley Scales of Infant Development.^12,14^ The follow-up studies by Juujärvi et al additionally reported two diagnoses of CP at two- and five-year follow up of the group for 44 children, with the authors concluding that the small sample size was not sufficient to detect differences in rare adverse neurodevelopmental outcomes.^12,13^ In their larger cohort study, 17 of 304 very preterm infants were diagnosed with CP at five years of age, with no significant association between paracetamol exposure and CP.^15^ Our cohort included more extreme preterm infants, with the group exposed to paracetamol having a mean gestational age of 26 weeks. This population is not only at higher risk of adverse neurodevelopmental outcomes, but may also be more susceptible to any adverse effects of early neonatal paracetamol exposure.

Paracetamol exposure is generally low in our unit, with PDA closure and post-operative (gastrointestinal surgery) analgesia being its primary uses. Preterm neonates on invasive ventilation with clinical features of PDA are evaluated by functional echocardiography (ECHO) and are treated if the ductal shunt is hemodynamically significant. The dosage for paracetamol is 15 mg/kg every six hours for three days (first course), administered intravenously or orally for infants tolerating trophic feeds. The second ECHO assessment is done within 24–48 h of the completion of the first course. A second course is given if there is a ductal patency, and paracetamol therapy for PDA beyond a second course is generally not used.^17^ In this study, 61% of infants exposed to paracetamol were treated for PDA. The second major indication for paracetamol use was following major surgery, such as a laparotomy. Post-operative paracetamol use was more commonly on an as-needed basis rather than regular regimented use as for PDA. In neonates, evidence shows that paracetamol has opioid-sparing effects for major pain syndromes and is effective for treating minor to moderate pain syndromes, with a good short-term safety profile.^3,24^ However, there remains limited data for its long-term safety, which forms the rationale for this study.

Our study has the following limitations. Firstly, the study is observational, and the baseline comparison between exposure and non-exposure was not balanced. Babies exposed to paracetamol were younger, smaller, and had more male infants. Besides, several neonatal morbidities were higher in the exposure group. We attempted to limit the effect of confounders on the association between exposure and the outcomes by regression analysis and were able to fit a few important confounders in the model. We also conceived the idea of propensity score analysis for robust matching; however, we could not perform it due to the small sample size.

Secondly, the various indications for paracetamol therapy were grouped as one exposure; however, the majority (61%) included infants treated with paracetamol for PDA. To overcome this limitation, we performed a subgroup analysis based on the dosing, which mostly captured infants treated with paracetamol for PDA (≥180 mg/kg) versus those for reasons other than PDA (<180 mg/kg). However, this is not an absolute distinction. Additionally, subgroup analyses may produce spurious results due to a lack of statistical power.

Thirdly, clinicians assessing neurodevelopmental outcomes were not blinded to paracetamol exposure. However, they were not aware of the exposure at the time of assessment so it is unlikely that this could have influenced their clinical judgement, and the study had not been conceived during this period. In addition, our study did not include those infants who died. As there were 34 deaths in this cohort during the study period, this may have missed those exposed to paracetamol and who may have had adverse neurodevelopmental outcomes if they survived.

Furthermore, the outcomes assessed were early neurodevelopmental outcomes at just 3–4 months corrected age. Yet these have been shown to be highly predictive of long-term developmental outcomes such as CP,^19^ and we believe these assessments are important for early diagnosis and management. Nonetheless, we intend to follow up this cohort of extreme preterm infants again at 24 months corrected age (18–24 months post term age), when they will be reviewed again at our Growth and Development Clinic and assessed using the Bayley Scales of Infant Development (IV Edition). We also acknowledge that most concerns after paracetamol exposure are related to behavioural concerns later in life, which will be evaluated at this subsequent clinic visit.

Lastly, we acknowledge the confidence of estimates for CP diagnosis and fidgety movements were wide and included a meaningful clinical benefit and harm as the frequency of these outcomes was low. While we did not perform power calculation a priori, a post-hoc analysis revealed a power of 45 percent, 9.2 percent and 89.6 percent for the outcomes early or high risk of CP, abnormal GMA, and abnormal HINE, respectively, suggesting the study being powered only for an abnormal HINE outcome.

## Conclusions

Exposure to paracetamol during the neonatal admission was not associated with significant differences in adverse early neurodevelopmental outcomes (early CP or high risk of CP diagnosis, HINE score, and fidgety GMA result) in a cohort of extreme preterm infants who survived and attended 3–4 month follow-up. While this is reassuring, the study was limited by its observational design and sample size. More prospective and long-term follow-up studies are required to confirm these findings and investigate further neurodevelopmental outcomes.

## Data Availability

De-identified group data will be available from authors on reasonable request.
